# Impact of antibiotic therapy on the development and response to treatment of immune checkpoint inhibitor-mediated diarrhea and colitis

**DOI:** 10.1186/s40425-019-0714-x

**Published:** 2019-09-05

**Authors:** Hamzah Abu-Sbeih, Lauren Nicholas Herrera, Tenglong Tang, Mehmet Altan, Anne-Maria P. Chaftari, Pablo C. Okhuysen, Robert R. Jenq, Yinghong Wang

**Affiliations:** 1 Department of Gastroenterology, Hepatology and Nutrition, The University of Texas MD Anderson Cancer Center, 1515 Holcombe Blvd, Houston, TX 77030 USA; 2 Department of Internal Medicine, Baylor College of Medicine, Houston, TX USA; 3 Department of Thoracic/Head and Neck Medical Oncology, The University of Texas MD Anderson Cancer Center, Houston, TX USA; 4 Department of Infectious Diseases, Infection Control and Employee Health, The University of Texas MD Anderson Cancer Center, Houston, TX USA; 5 Department of Genomic Medicine, The University of Texas MD Anderson Cancer Center, Houston, TX USA; 6 Minimally Invasive Surgery Center, The Second Xiangya Hospital of Central South University, Changsha, Hunan China

**Keywords:** Microbiome, Microbiota, Dysbiosis, Antibiotic therapy, Immune checkpoint inhibitor, ICI-mediated colitis, Colitis

## Abstract

**Background:**

The gut microbiome impacts the efficacy of immune checkpoint inhibitor (ICI) therapy and the development of ICI-mediated diarrhea and**/**or colitis (IMDC). Antibiotic therapy,especially that with anaerobic activity, has profound effects on the gut microbiome. Therefore, we sought to assess the effect of antibiotics on the development of IMDC.

**Methods:**

Patients who received ICI therapy from January 2016 to January 2018 were examined retrospectively. A Cox regression model was used to assess factors associated with overall survival.

**Results:**

A total of 826 patients were included. Of these patients, 51.6% received inhibitors of programmed cell death protein-1 or its ligand, 32.0% received inhibitors of cytotoxic T-lymphocyte-associated antigen-4, and 16.5% received a combination of the two. IMDC occurred in 52.5% of the patients after a median of 8 weeks. Overall, 569 patients (68.9%) received antibiotic therapy. Antibiotic use at any time was associated with reduced IMDC occurrence and recurrence rates but also with frequent hospitalization and intensive care unit admission for IMDC as well as increased IMDC severity. Compared with patients who received antibiotic therapy only before ICI therapy initiation, those receiving it after ICI had a higher IMDC rate and more often needed immunosuppressive therapy and hospitalization for IMDC. Antibiotics with anaerobic activity were included in 51% of the antibiotic therapy regimens and were associated with increased immunosuppressant use, hospitalization, intensive care unit admission for IMDC, and severe IMDC grades. Forty-one patients received empiric prophylactic antibiotic therapy at IMDC onset. These patients more often needed immunosuppressive therapy, intravenous steroids, and infliximab/vedolizumab; had more frequent and longer hospitalization for IMDC and higher IMDC grades; and more frequently had IMDC recurrence than did patients who did not receive antibiotic therapy at the time of IMDC symptom onset.

**Conclusions:**

Whereas antibiotic therapy appeared to be protective against IMDC onset, use of antibiotics, especially those with anaerobic activity, after ICI therapy was associated with increased risk of severe IMDC.

**Electronic supplementary material:**

The online version of this article (10.1186/s40425-019-0714-x) contains supplementary material, which is available to authorized users.

## Background

Immune checkpoint inhibitor (ICI) therapy has revolutionized cancer treatment. Its use has increased on a broad scale over the past decade, with promising outcomes. In parallel, the incidence of immunotherapy-related adverse events (irAEs) is on the rise, particularly immune-mediated diarrhea and/or colitis (IMDC), which is among the most common irAEs and frequently forces treatment discontinuation [1, 2]. IMDC can negatively affect the quality of life of an already vulnerable patient population. The incidence rate for gastrointestinal irAEs in patients given cytotoxic T-lymphocyte-associated antigen 4 (CTLA-4) inhibitors is as high as 30%, but it is lower in those given programmed cell death protein 1 (PD-1) and programmed death-ligand 1 (PD-L1) inhibitors (15%). When ICI therapy is combined, the rate can be as high as 55% [3]. Measures that minimize IMDC occurrence without compromising ICI efficacy are needed to optimize care for cancer patients.

The human body has about 100 trillion microbial cells, the majority of which are found in the gut and have physiologic implications [4]. Prior studies demonstrated that the gut microbiome plays a major role in the development and education of the immune system via several mechanisms, including cell signaling, interactions with antigen-presenting cells, and both T- and B-cell mediated immunity [5]. The gut microbiome also plays a role in modulation of the efficacy of ICI therapy [5]. Routy et al. [6] found that patients who received antibiotics before or soon after initiation of anti-PD-1 therapy had shorter progression-free and overall survival durations than did patients who did not receive antibiotics. They reported that higher levels of *Akkermansia muciniphila*, a gram-negative anaerobe, were associated with good outcomes in patients with lung or kidney cancer. This was confirmed by a study in which Routy and colleagues performed fecal microbiota transplantation (FMT) in tumor cell-inoculated mice using stool samples collected from patients that responded to ICI therapy. They found that the tumors in the mice were more sensitive to ICI therapy [6]. The intestinal microbiota composition also impacts IMDC, as FMT was successful in two patients with IMDC refractory to standard immunosuppressive therapy, resulting in symptomatic resolution and healing of colonic mucosal ulcerations [7]. In this study, *Akkermansia*, *Bacteroides*, and *Blautia* species had potential roles in alleviating IMDC.

Antibiotic therapy results in a decrease in the diversity and alteration of the microbiome composition (i.e., dysbiosis) for months or even years after treatment discontinuation [8]. Cancer patients are prone to infections due to their underlying malignancies, use of chemotherapy, immunosuppression, or stem cell transplantation. Physicians use intravenous and oral broad-spectrum antibiotics to treat these infections, resulting in dysbiosis. Given a lack of knowledge about the potential impact of antibiotic therapy on IMDC in cancer patients who receive ICIs, in the present study, we sought to investigate the effect of antibiotic therapy on the incidence and course of IMDC.

## Methods

### Patient population

This retrospective study was approved by the Institutional Review Board at The University of Texas MD Anderson Cancer Center. Adult cancer patients who received ICI therapy from January 2016 to January 2018 were included. The MD Anderson Pharmacy database was searched for details regarding ICI and antibiotic therapy in these patients. Afterward, a comprehensive chart review was conducted to extract variables of interest. IMDC was diagnosed after exclusion of other etiologies, including infectious colitis (Additional file 1: Table S1), graft-versus-host disease, and neutropenic colitis.

### Clinical characteristics

Collected information included patients’ demographic characteristics, clinical and oncologic histories, and clinical IMDC data. The demographic characteristics included age at the time of first ICI infusion, sex, and race/ethnicity. Comorbidities categorized according to the Charlson Comorbidities Index and cancer type and stage were documented. The type and duration of ICI treatment and nongastrointestinal irAEs were also collected. Information regarding the specific antibiotic therapy prescribed to each patient within the study time window (i.e., from 3 months prior to ICI initiation to the onset of IMDC or 3 months after the last ICI therapy administration if the patient did not have IMDC) was recorded. Patients were categorized into three groups according to the timing of antibiotic therapy: 1) only before ICI therapy initiation, 2) only after ICI therapy, and 3) both before and after ICI therapy. Also, regarding the coverage of antibiotic therapy, patients were categorized as those receiving antibiotics with anaerobic activity (Additional file 1: Table S2) or those receiving antibiotics with aerobic activity only.

### IMDC characteristics

Data pertaining to IMDC that were analyzed were the time from ICI therapy initiation to IMDC onset, duration of symptoms, peak grades of diarrhea and colitis, treatments, and outcomes. IMDC was graded using the Common Terminology Criteria for Adverse Events (version 5.0) [9]. Colitis was graded when there is either clinical symptoms suggestive of colitis (i.e. abdominal pain, abdominal distension, fever, blood or mucus per stool) or diagnostic features by laboratory, imaging, or endoscopic modalities. If only diarrhea was present without colitis features, then grade of diarrhea was provided without colitis grading (Additional file 1: Table S3). Treatment of IMDC consisted of immunosuppressants (i.e., steroids with or without infliximab and vedolizumab) or symptomatic support only. The cumulative duration of steroid-based treatment was measured. Requirement of hospitalization and intensive care unit (ICU) admission because of IMDC was documented. Additionally, the cumulative duration of IMDC-associated hospitalizations was reported. Furthermore, recurrence of IMDC after complete discontinuation of immunosuppressive therapy and any IMDC-related complications (e.g., colonic perforation) were recorded. The overall survival duration was defined as the time from ICI therapy initiation to last clinical encounter or death.

### Statistical analyses

Continuous variables were presented using means and standard deviations (SDs) or medians and interquartile ranges (IQRs). Categorical variables were presented using frequencies and percentages. Fisher exact and χ^2^ tests were used to compare categorical variables. The Wilcoxon rank sum and Kruskal-Wallis tests were used to compare continuous variables. Kaplan-Meier curves and log-rank tests were used to estimate and compare overall survival durations between subgroups. A multivariate Cox model was used to assess the independent impact of each variable on overall survival. All statistical tests were two-sided. *P* values of up to 0.05 were considered significant. Statistical analyses were performed using the SAS (version 9.4; SAS Institute) and SPSS (version 24.0; IBM) software programs.

## Results

### Study population

**Fig. 1:**
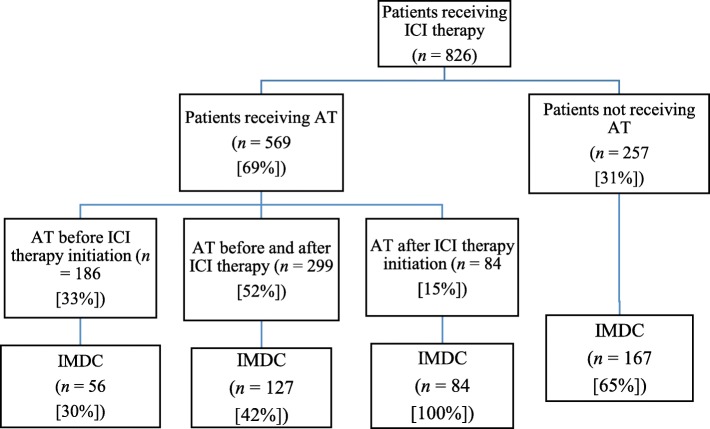
Schematic of study population according to ICI therapy, antibiotic therapy (AT) and IMDC

A total of 826 cancer patients were included (Fig. 1): 426 (51.6%) received anti-PD-1/PD-L1 therapy, 264 (32.0%) received anti-CTLA-4 therapy, and 136 (16.5%) received a combination of the two. Their median age was 62 years (IQR, 52–70 years), and most of them were male (*n* = 524 [63.4%]). Melanoma was the most common malignancy (*n* = 347 [42%]). The majority of the patients had stage IV malignancies (*n* = 624 [88%]) (Table 1).

**Table 1:** Clinical characteristics of the study population (*n* = 826)

Characteristic	*n* (%)
Median age, years (IQR)	62 (52–70)
Male sex	524 (63.4)
Non-Hispanic white	704 (85.2)
Comorbidities	408 (49.4)
Smoking	390 (47.2)
Cancer type	
Melanoma	347 (42.0)
Solid tumor	363 (43.9)
Hematologic	116 (14.0)
Cancer stage (*n* = 709)	
III	85 (12.0)
IV	624 (88.0)
ICI type	
Anti-CTLA-4	264 (32.0)
Anti-PD-1/L1	426 (51.6)
Combination	136 (16.5)
Median duration of ICI therapy, days (IQR)	56 (22–116)
IMDC	434 (52.5)
Median time to IMDC onset, weeks (IQR)	8 (4–15)
Median duration of IMDC symptoms, days (IQR)	9 (3–20)
Grade of colitis (*n* = 319)	
1	74 (23.2)
2	150 (47.0)
3	84 (26.3)
4	11 (3.4)
Grade of diarrhea (*n* = 434)	
1	129 (29.7)
2	120 (27.6)
3	164 (37.8)
4	21 (4.8)
Treatment of IMDC (*n* = 434)	
Immunosuppressants	276 (63.6)
Symptomatic only	158 (36.4)
Infliximab and**/**or vedolizumab add-on	83 (10.0)
Antibiotic therapy	
No	257 (31.1)
Yes	569 (68.9)
Before ICI therapy only	186 (32.7)
After ICI therapy and before IMDC onset	84 (14.8)
Both	299 (52.5)
Use of antibiotics with antianaerobic activity	288 (50.6)
Recurrence of IMDC	83 (10.0)
Colon perforation	7 (1.0)
Nongastrointestinal irAEs	312 (37.8)

### Clinical IMDC data

IMDC developed in 434 patients (52.5%). Their median time to onset of IMDC was 8 weeks (IQR, 4–15 weeks), with a median duration of symptoms of 9 days (IQR, 3–20 days). The most common grade of colitis was 2 (*n* = 150 of 319 patients having colitis [47.0%]), whereas the most common grade of diarrhea was 3 (*n* = 164 of 434 patients having diarrhea [37.8%]). IMDC treatment consisted of immunosuppressants in 276 patients (63.6%), and symptomatic support in 158 patients (36.4%) (Table 1).

### Overview of antibiotic therapy

Of the 826 study patients, 569 (68.9%) received antibiotic therapy (Table 1). Of these 569 patients, 299 (52.5%) received antibiotics both before and after initiation of ICI therapy, 186 (32.7%) received antibiotics before initiation of ICI therapy only, and 84 (14.8%) received antibiotics after initiation of ICI therapy only. Empirical use of antibiotic without an identifiable source was the most common indication for antibiotic use, followed by prophylactic use without signs of infection (Additional file 1: Table S4). Overall, patients who received antibiotics had lower IMDC occurrence (*p* < 0.001) and recurrence (*p* = 0.025) rates than did patients without antibiotic exposure (Table 2). However, more IMDC patients who received antibiotics needed hospitalization (*p* < 0.001) or ICU admission (*p* = 0.038) or had severe colitis (*p* = 0.022) than did patients who did not receive antibiotics.

**Table 2:** Clinical features of the study patients according to use of antibiotic therapy

Feature	Antibiotic therapy (*n* = 569)	No antibiotic therapy (*n* = 257)	*p*
IMDC, *n* (%)	267 (46.9)	167 (65.0)	< 0.001
Mean duration of IMDC symptoms, days (SD)	32 (150)	18 (102)	0.290
Hospitalization, *n* (%)	168 (62.9)	63 (37.7)	< 0.001
Mean duration of hospitalization, days (SD)	8 (7)	7 (5)	0.143
ICU admission, *n* (%)	10 (1.8)	1 (0.3)	0.038
Grade of colitis, *n* (%)			0.022
1	42 (20.7)	32 (27.6)	
2	90 (44.3)	60 (51.7)	
3	65 (32.0)	19 (16.4)	
4	6 (3.0)	5 (4.3)	
Grade of diarrhea, *n* (%)			0.098
1	77 (28.8)	52 (31.1)	
2	69 (25.8)	51 (30.5)	
3	103 (38.3)	61 (36.5)	
4	18 (6.7)	3 (1.8)	
Mean duration of steroid administration, days (SD)	52 (44)	63 (79)	0.147
Infliximab/vedolizumab administration, *n* (%)	52 (19.5)	31 (18.6)	0.900
Recurrence of IMDC, *n* (%)	42 (15.4)	41 (24.6)	0.025

Of the patients given antibiotics, 51% received antibiotics with anaerobic activity. When we compared the patients given anaerobic and aerobic antibiotic agents, we noticed that anaerobic antibiotic therapy was associated with increased rates of hospitalization (*p* < 0.001) and ICU admission for IMDC (*p* = 0.002), IMDC grade (*p* = 0.004), and requirement of immunosuppressive therapy (*p* = 0.03) (Table 3).

**Table 3:** Clinical features of patients who received anaerobic and aerobic antibiotic therapy (No. of patients who received antibiotic = 569)

Feature	Anaerobic (*n* = 288)	Aerobic (*n* = 281)	*p*
Indication for antibiotic, *n* (%)			< 0.001
Upper respiratory infection	12 (4.2)	9 (3.2)	
Lower respiratory infection	22 (7.6)	16 (5.7)	
Gastrointestinal infection	37 (12.8)	31 (11.0)	
Urinary tract infection	21 (7.3)	41 (14.6)	
Skin/Soft tissue infection	20 (6.9)	23 (8.2)	
Sepsis and bacteremia	7 (2.4)	4 (1.4)	
Fever of unknown origin/empirical coverage	101 (35.1)	65 (23.1)	
Prophylaxis	25 (8.7)	65 (23.1)	
Multiple infections	40 (13.9)	14 (5.0)	
Not recorded	3 (1.0)	13 (4.6)	
IMDC, *n* (%)	145 (50.3)	122 (43.4)	0.093
Immunosuppressive therapy for IMDC, *n* (%)	102 (35.4)	71 (25.3)	0.030
Median time to IMDC onset, weeks (IQR)	8 (4–15)	7 (4–13)	0.075
Median duration of IMDC symptoms, days (IQR)	9 (4–19)	10 (4–20)	0.118
Hospitalization, *n* (%)	106 (73.1)	62 (50.8)	< 0.001
Median duration of hospitalization, days (IQR)	6 (3–10)	6 (3–10)	0.111
ICU admission, *n* (%)	10 (6.9)	0	0.002
Grade of colitis, *n* (%)			0.004
1	16 (13.2)	26 (31.7)	
2	59 (48.8)	31 (37.8)	
3	40 (33.1)	25 (30.5)	
4	6 (5.0)	0	
Grade of diarrhea, *n* (%)			0.087
1	38 (26.2)	39 (32.0)	
2	33 (22.8)	36 (29.5)	
3	60 (41.4)	43 (35.2)	
4	14 (9.7)	4 (3.3)	
Mean calprotectin level (SD)	352 (348)	181 (137)	0.083
Median duration of steroid administration, days (IQR)	37 (20–61)	45 (27–70)	0.355
Intravenous steroid administration, *n* (%)	61 (63.5)	38 (55.1)	0.334
Infliximab/vedolizumab administration, *n* (%)	28 (19.3)	24 (19.7)	1.000
Recurrence of IMDC, *n* (%)	25 (17.2)	17 (13.9)	0.503
Colon perforation, *n* (%)	4 (2.8)	0	0.128

#### Patients who received anti-CTLA-4

Additional file 1: Table S5.A summarizes the clinical features of patients who received antibiotics. The rate of IMDC was lower in patients who received antibiotic therapy compared with those who did not (*p* = 0.002). Moreover, antibiotic use was associated with more frequent hospitalizations (*p* < 0.001) and higher grades of colitis (*p* = 0.011). Antibiotics with anaerobic activity were associated with higher rates of IMDC (*p* = 0.021), more frequent requirement for immunosuppressive therapy (*p* = 0.014), more frequent hospitalizations (*p* = 0.002), higher grades of colitis (*p* = 0.009), and higher levels of fecal calprotectin (*p* = 0.010) (Additional file 1: Table S5.B).

#### Patients who received anti-PD-1/L1

Patients who received antibiotics had lower rates of IMDC (*p* = 0.001) and IMDC recurrence (*p* = 0.045) (Additional file 1: Table S6.A). Antibiotic use was associated with more frequent hospitalizations (*p* < 0.001). Likewise, antibiotics with anaerobic activity were associated with more frequent hospitalizations (*p* = 0.046) and ICU admissions (*p* = 0.027), as well as more requirement for IV corticosteroids (*p* = 0.017) (Additional file 1: Table S6.B).

### Timing of antibiotic therapy

Among the patients who had IMDC, 41 received empiric prophylactic antibiotic therapy at the time of IMDC onset without laboratory confirmation of an active infection, whereas 393 did not (Table 4). For these 41 patients, the median time from IMDC symptoms onset to antibiotic treatment was 4 days (IQR, 1–8 days), and median time from hospitalization to antibiotics was 7 days (IQR, 4–16 days). Among these patients, 21 patients had colitis confirmed on imaging (10 had diffuse colitis and 11 had segmental colitis), with no reported serious complications related to colitis, e.g. abscess, perforation, toxic colitis, or megacolon. Endoscopically confirmed colitis was evident in 21 patients (11 had extensive colitis beyond splenic flexure), among which, 11 had ulcerations and 10 had non-ulcerative inflammation. Patients receiving empiric antibiotic therapy had higher IMDC grades, more frequent hospitalizations (*p* < 0.001), longer hospital stays (*p* = 0.003), more frequent need for treatment with immunosuppressants (*p* < 0.001) and infliximab/vedolizumab (*p* < 0.001), and a higher IMDC recurrence rate (*p* = 0.038) than did patients who did not receive antibiotic therapy at the time of IMDC onset.

**Table 4:** Clinical features of the study patients according to use of empiric antibiotic therapy at IMDC onset (No. of patients with IMDC = 434)

Feature	Antibiotic therapy at IMDC onset (*n* = 41)	No antibiotic therapy (*n* = 393)	*p*
Immunosuppressive therapy for IMDC, *n* (%)	41 (100.0)	235 (59.8)	< 0.001
Median duration of IMDC symptoms, days (IQR)	17 (8–32)	8 (3–19)	0.888
Hospitalization, *n* (%)	41 (100)	190 (48.3)	< 0.001
Median duration of hospitalization, days (IQR)	9 (4–15)	5 (3–8)	0.003
ICU admission, *n* (%)	3 (7.3)	8 (2.0)	0.076
Grade of colitis, *n* (%)			0.001
1	6 (14.6)	68 (24.5)	
2	12 (29.3)	138 (49.6)	
3	22 (53.7)	62 (22.3)	
4	1 (2.4)	10 (3.6)	
Grade of diarrhea, *n* (%)			< 0.001
1	4 (9.8)	125 (31.8)	
2	8 (19.5)	112 (28.5)	
3	24 (58.5)	140 (35.6)	
4	5 (12.2)	16 (4.1)	
Median duration of steroid administration, days (IQR)	39 (23–64)	45 (23–70)	0.496
Intravenous steroid administration, *n* (%)	35 (85.4)	112 (50.2)	< 0.001
Infliximab/vedolizumab administration, *n* (%)	18 (43.9)	65 (16.5)	< 0.001
Recurrence of IMDC, *n* (%)	13 (31.7)	70 (17.8)	0.038
Colon perforation, *n* (%)	1 (2.4)	6 (1.5)	0.503

When we separated the duration of antibiotic use relative to ICI therapy initiation and IMDC onset, the 84 patients exposed to antibiotics after initiation of ICI therapy and before IMDC onset had a higher rate of IMDC occurrence (*p* < 0.001) and more often needed hospitalization (*p* = 0.044) and immunosuppressive therapy (*p* < 0.001) than did the 186 patients who received antibiotics before ICI therapy and the 299 patients exposed to antibiotics both before and after ICI therapy (Table 5).

**Table 5:** Clinical features of the study patients according to timing of antibiotic administration

Feature	Before ICI therapy (*n* = 186)	After ICI therapy and before IMDC onset (*n* = 84)	Both (*n* = 299)	*p*
Indication for antibiotic, *n* (%)				0.015
Upper respiratory infection	4 (2.2)	3 (3.6)	14 (4.7)	
Lower respiratory infection	16 (8.6)	3 (3.6)	19 (6.4)	
Gastrointestinal infection	12 (6.5)	20 (23.8)	36 (12.0)	
Urinary tract infection	19 (10.2)	8 (9.5)	35 (11.7)	
Skin/Soft tissue infection	13 (7.0)	9 (10.7)	21 (7.0)	
Sepsis and bacteremia	5 (2.7)	3 (3.6)	3 (1.0)	
Fever of unknown origin/empirical coverage	52 (28.0)	20 (23.8)	94 (31.4)	
Prophylaxis	35 (18.8)	9 (10.7)	46 (15.4)	
Multiple infections	21 (11.3)	9 (10.7)	24 (8.0)	
Not recorded	9 (4.8)	0 (0.0)	7 (2.3)	
IMDC, *n* (%)	56 (30.1)	84 (100.0)	127 (42.5)	< 0.001
Immunosuppressive therapy for IMDC, *n* (%)	33 (17.7)	65 (77.4)	75 (25.1)	< 0.001
Median time to IMDC onset, weeks (IQR)	11 (8–16)	9 (5–14)	5 (2–11)	0.134
Median duration of IMDC symptoms, days (IQR)	10 (4–21)	10 (4–20)	10 (4–19)	0.801
Hospitalization, *n* (%)	33 (58.9)	62 (73.8)	73 (57.5)	0.044
Median duration of hospitalization, days (IQR)	5 (3–8)	7 (3–12)	6 (3–9)	0.234
ICU admission, *n* (%)	1 (1.8)	3 (3.6)	6 (4.7)	0.625
Grade of colitis, *n* (%)				0.413
1	12 (30.0)	9 (12.5)	21 (23.1)	
2	17 (42.5)	35 (48.6)	38 (41.8)	
3	10 (25.0)	25 (34.7)	30 (33.0)	
4	1 (2.5)	3 (4.2)	2 (2.2)	
Grade of diarrhea, *n* (%)				0.976
1	17 (30.4)	21 (25.0)	39 (30.7)	
2	14 (25.0)	24 (28.6)	31 (24.4)	
3	22 (39.3)	33 (39.3)	48 (37.8)	
4	3 (5.4)	6 (7.1)	9 (7.1)	
Intravenous steroid administration, *n* (%)	14 (46.7)	41 (65.1)	44 (61.1)	0.232
Infliximab/vedolizumab administration, *n* (%)	6 (10.7)	22 (26.2)	24 (18.9)	0.075
Recurrence of IMDC, *n* (%)	8 (14.3)	16 (19.0)	18 (14.2)	0.601
Colon perforation, *n* (%)	1 (1.8)	1 (1.2)	2 (1.6)	1.000

### Multivariate logistic regression of IMDC risk

Anti-CTLA-4 therapy was associated with higher risk of IMDC (*p* < 0.001) (Additional file 1: Table S7). By contrast, antibiotic therapy (*p* < 0.001) with anaerobic activity (*p* < 0.001) was associated with lower risk of IMDC.

### Survival analyses

Univariate Cox survival analyses demonstrated that antibiotic exposure overall (*p* < 0.001) and exposure to antibiotics with anaerobic activity specifically (*p* < 0.001) were associated with poor overall survival rates (Additional file 1: Figure S1 and Additional file 1: Figure S2). Also, timing of antibiotic therapy after ICI therapy initiation was associated with poor overall survival (*p* = 0.013). Other factors associated with poor overall survival included advanced age, increased calprotectin levels, and stage IV cancer. Longer duration of IMDC symptoms, onset of IMDC, duration of anti-CTLA-4 therapy, and duration from ICI therapy initiation to IMDC onset were correlated with better overall survival (Additional file 1: Table S8). In multivariate Cox regression model, stage IV cancer and anaerobic antibiotic therapy were associated with poor overall survival rates (*p* = 0.038 and *p* = 0.007, respectively). On the other hand, IMDC occurrence was associated with better overall survival rates than in patients without IMDC (*p* < 0.001) (Table 6).

**Table 6:** Multivariate Cox regression analysis of overall survival in the study population

Characteristic	HR (95% CI)	*p*
ICI type		
Anti-PD-1/PD-L1	Reference	
Anti-CTLA-4	0.85 (0.56–1.28)	0.434
Combination	0.78 (0.58–1.05)	0.097
Stage IV cancer	1.63 (1.03–2.60)	0.038
IMDC	0.45 (0.34–0.61)	< 0.001
Anaerobic antibiotic therapy	1.44 (1.11–1.87)	0.007

*Abbreviations*: *HR* Hazard ratio, *CI* Confidence interval

## Discussion

IMDC is among the most common severe toxic effects that lead to ICI therapy discontinuation. Nonetheless, the underlying pathogenesis of IMDC remains unclear. Recent studies suggested a role of the gut microbiome in the development of IMDC as well as in the response of IMDC to treatment, as it can impact the immune system. In both animal and human studies, FMT has been beneficial for recovery from IMDC. [7, 10] Given that antibiotic therapy is frequently used in cancer patients and given its impact on the gut microbiome, we evaluated the association between antibiotic therapy and the development and severity of IMDC in cancer patients receiving ICI therapy. We found that use of antibiotics, especially those with anaerobic activity and when given after ICI therapy initiation, was associated with an increased risk of more severe IMDC. Moreover, prophylactic antibiotic therapy at the time of IMDC onset correlated with worse IMDC course. Finally, treatment with antibiotics having anaerobic activity was associated with poor overall survivals. Of note, further prospectively designed studies are needed to investigate the associations in the current report.

In this study, we specifically investigated the association between antibiotics and IMDC according to three factors: overall exposure to antibiotic therapy, spectrum of antibiotic therapy coverage, and timing of antibiotic therapy relative to ICI therapy initiation. Exposure to antibiotics was associated with decreased rates of occurrence and recurrence of IMDC, but when present, IMDC was likely to be severe. Therefore, we looked for potentially concealed factors leading to this observation and thus separately examined antibiotic therapy according to the microbial spectrum of antibiotic activity and time given. We observed that the rate of IMDC was slightly higher in patients given antibiotics with anaerobic activity and those who received antibiotic therapy after ICI therapy initiation. Likewise, the severity of IMDC in these patients was higher. Therefore, the timing and microbial spectrum of activity of antibiotic therapy are more impactful than use of the therapy in general.

Given that anti-CTLA-4 therapy has a distinct mechanism of action and toxicity profile than anti-PD-1/L1, we performed separate analyses to assess the association between antibiotics and IMDC among each class. The findings of these analyses revealed similar conclusions to those when performed together. The rate of IMDC in the current study was slightly higher than what was reported as the overall incidence of any grade diarrhea or colitis, likely because almost half of the current cohort received CTLA-4 inhibitors. In addition, we captured both diarrhea and colitis as separate entities in the beginning but when reporting the rate of IMDC it was a combination of both. In a review by Kumar et al., the rate of any grade diarrhea was reported as up to 54%, especially in patients receiving anti-CTLA-4 therapy.

Interestingly, as a well-known risk factor for gut dysbiosis, antibiotic therapy with anaerobic activity was administered in half of our cohort and was associated with increased hospitalization and ICU admission for IMDC, increased grades of IMDC, and increased requirement of immunosuppressive therapy. Treatment with anaerobic antibiotics can theoretically disrupt the gut microbiota substantially given that 95% of the normal gut bacterial composition is anaerobes according to the gut’s known taxonomic composition [11]. Certain anaerobes, such as *A*. *muciniphila*, are beneficial in that they attenuate colitis. [6] Therefore, we hypothesized that the unfavorable gut microbiota changes resulting from the use of antibiotics with anaerobic activity can contribute to altered regulation of the immune system, facilitating IMDC development. In contrast, we did not observe a strong association between aerobic antibiotic therapy and IMDC.

Importantly, a portion of our cohort received empiric antibiotic therapy at the onset of IMDC without confirmation of an active infection. The infectious workup, including multiplex study, in these patients was negative for infection at time of IMDC onset. Similarly, endoscopy with biopsy was performed in a few of them and confirmed IMDC. This approach was pursued more often in patients with severe IMDC disease course reflected by high grades of IMDC, frequent hospitalization with long hospital stays, and frequent use of immunosuppressive therapy, including intravenous steroids and infliximab/vedolizumab. Of note, these patients were likely to experience recurrence of IMDC. The causative correlation between severity of IMDC and antibiotic use (i.e. antibiotic use led to more severe IMDC versus severe IMDC symptoms led to more frequent use of antibiotics) could not be determined based on findings of this study given its retrospective design and the presence of most severity indicators before antibiotic treatment initiation. There was no beneficial impact from empiric antibiotic use on IMDC outcomes. These observations stress the importance of careful evaluation of patients for active infection before starting antibiotic therapy. Before implementation of the current treatment guidelines and with limited awareness of these toxic effects, physicians commonly used antibiotic therapy empirically to manage diarrhea. Our findings reinforce the recommendation of avoiding empiric antibiotic therapy in patients who suffer from gastrointestinal symptoms following immunotherapy unless they have clear infections.

Because patients may receive antibiotics at different time points during their cancer treatment courses, we dissected the time-specific effects of antibiotic therapy on IMDC. Among the three groups of patients receiving antibiotic therapy, those given antibiotics after ICI therapy initiation experienced the worst outcomes, including the highest rate of IMDC occurrence and requirement of hospitalization and immunosuppressive therapy. In contrast, patients with antibiotic exposure before ICI therapy initiation or both before and after it did not have comparable results. ICI therapy may have contributed to alteration of the gut microbiota or even had a synergistic effect on the development of more significant dysbiosis with subsequent antibiotic use. This is similar to findings from a previous study by Dubin et al. [12] They examined patients with melanoma treated with an anti-CTLA-4 regimen who underwent longitudinal follow-up. Stool metagenomic analysis in patients who had colitis demonstrated dramatic changes compared with those who did not have it. This certainly added complexity to attempts to manipulate the gut microbiome and subsequently affected the immune response. However, the underlying mechanism of reversal of this negative impact in the patients with antibiotic exposure before or both before and after ICI therapy initiation, especially the latter, was not clear. Whether different sequential orders of antibiotic and ICI exposure have different effects needs further clarification using microbiome analyses.

Given the limited available information about whether fecal microbiome plays a critical role in the clinical response of IMDC by affecting T-cell function in the tumor microenvironment, [5, 6] our group performed FMT as a novel treatment of IMDC refractory to immunosuppressive therapy [7]. Of note, FMT led to resolution of clinical symptoms in IMDC patients within 2 weeks, with almost complete healing of the colonic mucosa. Metagenomic analysis of stool samples from these patients demonstrated successful engraftment of donor stool microbiota, and among these organisms, *Akkermansia*, *Bacteroides*, and *Blautia* spp. were significantly increased. The change**s** in the patient’s immune profile according to colon biopsy suggested a consistent pattern of reversal of the inflammatory process, with decreases in the numbers of CD8 T-cell subtypes and persistence or increases in the numbers of CD4+ FoxP3+ cells. Findings of both the present study and our previous FMT study argue with the role of the microbiome in modulating IMDC.

In addition, our survival analysis showed that IMDC was associated with favorable overall survival, which was consistent with our previous studies [13]. Nonetheless, other studies reported conflicting results regarding this observation [14, 15]. In contrast, anaerobic antibiotic therapy was correlated with a poor overall survival rate, probably due to significant gut dysbiosis. Nonetheless, underlying malignancy progression and systemic immunosuppression may have dictated more frequent antibiotic use, which will lead to worse outcomes. This conclusion was supported by a study by Gopalakrishnan et al., [16] who showed that certain microbiome patterns were associated with different cancer responses to ICI regimens. In addition, FMT with stool collected from cancer patients who were ICI therapy responders produced better tumor regression than did FMT with stool from nonresponders in mice inoculated with tumor cells. Determining whether providing healthy stool microbiota to cancer patients prior to ICI therapy initiation can prevent the onset of IMDC and boost the effect of ICIs to improve overall cancer response still needs further investigation.

Our study had some limitations. The retrospective study design may have limited the availability and accuracy of the details of the antibiotic therapy regimens, specifically when antibiotic therapy was prescribed at an outside institution. Furthermore, the decision to give antibiotic therapy at our institution was made based on the clinical judgment of the treating physician, and there is no universal algorithm that was used for patients receiving ICI therapy. This random selection manner alongside with the current findings of our study stress the need for a systematic guidance regarding the appropriate indications for antibiotic therapy in patients receiving ICI therapy, preferably a collaborative effort from both oncologists and infectious disease physicians. Also, we did not assess whether the presence of other confounding factors, for instance, probiotic remedies and diet, impacted the gut microbiota. Furthermore, we were unable to collect data regarding confirmation of specific infections in our cohort due to the retrospective nature of the study. The results of our survival analysis may have been confounded by the combination of various cancer types, use of different ICIs, and patients’ functional status. Finally, we did not analyze the gut microbiome composition**s** in this cohort. Therefore, our conclusions are associations.

## Conclusions

Treatment with antibiotics having anaerobic activity was associated with an increased risk of IMDC with a more severe disease course, especially when given after ICI therapy. Empiric antibiotic use in patients with gastrointestinal symptoms receiving ICI therapy should be considered only when the suspicion for infectious etiology is high. Patients who received anaerobic antibiotic therapy had worse survival rates than did those who did not. However, this finding should be interpreted with caution. We suspect that dysbiosis of the gut microbiota is a sequela of antibiotic therapy administered with ICI therapy. Due to the indirect nature of our conclusions, further stool metagenomic analyses are needed to clarify the role of antibiotics and the gut microbiome in the development of IMDC and their influence on IMDC outcome. Similarly, prospective studies are warranted to determine the effect of antibiotic therapy on overall survival.

## Additional file

Additional file 1: **Table S1.** Pathogens tested for by gastrointestinal multiplex laboratory testing at our institution. **Table S2.** Antibiotics with anti-anaerobic activity administered to study patients. **Table S3.** Common terminology Criteria for Adverse Events grading for diarrhea and colitis. **Table S4.** Indications for antibiotic use. **Table S5. A.** Clinical features of patients who received anti-CTLA-4 therapy according to use of antibiotic therapy. **Table S5. B.** Clinical features of patients who received anaerobic and aerobic antibiotic therapy among patients who received anti-CTLA-4 therapy. (No. of patients who received antibiotics = 270). **Table S6. A.** Clinical features of patients who received anti-PD-1/L1 therapy according to use of antibiotic therapy. **Table S6. B** Clinical features of patients who received anaerobic and aerobic antibiotic therapy among patients who received anti-PD-(L)1 therapy. (No. of patients who received antibiotics = 299). **Table S7.** Multivariate logistic regression analysis of risk of IMDC. **Table S8.** Univariate Cox regression analysis of overall survival in the study population. **Figure S1.** Kaplan-Meier curve for overall survival of patients who did and did not receive antibiotic therapy. **Figure S2.** Kaplan-Meier curves for overall survival of patients who did and did not receive antibiotic therapy with antianaerobic acivity. (DOCX 48 kb)

## Data Availability

The datasets used and analyzed during the current study are available from the corresponding author on reasonable request.

## Abbreviations

CTLA-4: Cytotoxic T-lymphocyte-associated antigen 4
FMT: Fecal microbiota transplantation
ICI: Immune checkpoint inhibitor
ICU: Intensive care unit
IMDC: Immune-mediated diarrhea and colitis
IQR: Interquartile range
irAE: Immunotherapy-related adverse event
PD-1: Programmed cell death protein 1
PD-L1: Programmed death-ligand 1
SD: Standard deviation
