# Correction to: Impact of antibiotic therapy on the development and response to treatment of immune checkpoint inhibitor-mediated diarrhea and colitis

**DOI:** 10.1186/s40425-019-0832-5

**Published:** 2019-12-17

**Authors:** Hamzah Abu-Sbeih, Lauren Nicholas Herrera, Tenglong Tang, Mehmet Altan, Anne-Marie Chaftari, Pablo C. Okhuysen, Robert R. Jenq, Yinghong Wang

**Affiliations:** 1 Department of Gastroenterology, Hepatology and Nutrition, The University of Texas MD Anderson Cancer Center, 1515 Holcombe Blvd, Houston, TX 77030 USA; 2 Department of Internal Medicine, Baylor College of Medicine, Houston, TX USA; 3 Department of Thoracic/Head and Neck Medical Oncology, The University of Texas MD Anderson Cancer Center, Houston, TX USA; 4 Department of Infectious Diseases, Infection Control and Employee Health, The University of Texas MD Anderson Cancer Center, Houston, TX USA; 5 Department of Genomic Medicine, The University of Texas MD Anderson Cancer Center, Houston, TX USA; 6 Minimally Invasive Surgery Center, The Second Xiangya Hospital of Central South University, Changsha, China

**Correction to: J ImmunoTher Cancer**

**https://doi.org/10.1186/s40425-019-0714-x**

Following publication of the original article [1], the authors have reported that an author’s name has been incorrectly spelled: the correct given name is Anne-Marie (instead of Anne-Maria P) and family name is Chaftari.
