# Supplementary material for: Impact of antibiotic therapy on the development and response to treatment of immune checkpoint inhibitor-mediated diarrhea and colitis
Source: J Immunother Cancer. 2019 Sep 5;7:242. doi: 10.1186/s40425-019-0714-x (PMC6729015; doi:10.1186/s40425-019-0714-x)
Supplement: Supplementary file 1 — Table S1. Pathogens tested for by gastrointestinal multiplex laboratory testing at our institution. Table S2. Antibiotics with anti-anaerobic activity administered to study patients. Table S3. Common terminology Criteria for Adverse Events grading for diarrhea and colitis. Table S4. Indications for antibiotic use. Table S5. A. Clinical features of patients who received anti-CTLA-4 therapy according to use of antibiotic therapy. Table S5. B. Clinical features of patients who received anaerobic and aerobic antibiotic therapy among patients who received anti-CTLA-4 therapy. (No. of patients who received antibiotics = 270). Table S6. A. Clinical features of patients who received anti-PD-1/L1 therapy according to use of antibiotic therapy. Table S6. B Clinical features of patients who received anaerobic and aerobic antibiotic therapy among patients who received anti-PD-(L)1 therapy. (No. of patients who received antibiotics = 299). Table S7. Multivariate logistic regression analysis of risk of IMDC. Table S8. Univariate Cox regression analysis of overall survival in the study population. Figure S1. Kaplan-Meier curve for overall survival of patients who did and did not receive antibiotic therapy. Figure S2. Kaplan-Meier curves for overall survival of patients who did and did not receive antibiotic therapy with antianaerobic acivity. (DOCX 48 kb) [file 40425_2019_714_MOESM1_ESM.docx]

| Campylobacter |  |  |
| --- | --- | --- |
| C difficile DNA |  |  |
| Plesiomonas shigelloides |  |  |
| Salmonella |  |  |
| Vibrio |  |  |
| Vibrio cholerae |  |  |
| Yersinia enterocolitica |  |  |
| Enteroaggregative E. coli |  |  |
| Enteropathogenic E. coli |  |  |
| Enterotoxigenic E. coli |  |  |
| Shiga-like toxin-producing E. coli |  |  |
| E. coli O157 |  |  |
| Shigella/Enteroinvasive E. coli |  |  |
| Cryptosporidium |  |  |
| Cyclospora cayetanensis |  |  |
| Entamoeba histolytica |  |  |
| Giardia lamblia |  |  |
| Adenovirus F 40/41 |  |  |
| Astrovirus |  |  |
| Norovirus GI/GII |  |  |
| Rotavirus A |  |  |
| Sapovirus (I, II, IV) |  |  |

**Table S1** Pathogens tested for by gastrointestinal multiplex laboratory testing at our institution

**Table S2** Antibiotics with anti-anaerobic activity administered to study patients

| Amoxicillin-clavulanic acid |
| --- |
| Ampicillin-sulbactam |
| Cefdinir  Cefoxitin  Cefotetan |
| Clindamycin |
| Delafloxacin |
| Doripenem |
| Eravacycline |
| Ertapenem |
| Imipenem |
| Meropenem |
| Metronidazole |
| Moxifloxacin |
| Omadacycline |
| Penicillin |
| Piperacillin-tazobactam |
| Tigecycline |

**Table S3** Common terminology Criteria for Adverse Events grading for diarrhea and colitis.

|  | Grade | | | | |
| --- | --- | --- | --- | --- | --- |
| Adverse Events | 1 | 2 | 3 | 4 | 5 |
| Diarrhea | Increase of <4 stools per day over baseline; mild increase in ostomy output compared to baseline | Increase of 4 - 6 stools per day over baseline; moderate increase in ostomy output compared to baseline | Increase of ≥7 stools per day over baseline; incontinence; hospitalization indicated; severe increase in ostomy output compared to baseline; limiting self-care ADL | Life-threatening consequences; urgent intervention indicated | Death |
| Colitis | Asymptomatic; clinical or diagnostic observations only; intervention not indicated | Abdominal pain; mucus or blood in stool | Severe abdominal pain; peritoneal signs | Life-threatening consequences; urgent intervention indicated | Death |

ADL: activity of daily living

**Table S4** Indications for antibiotic use.

| **Indication** | **No. of patients** |
| --- | --- |
| Upper respiratory infection | 21 (3.7) |
| Lower respiratory infection | 38 (6.7) |
| Gastrointestinal infection | 68 (12.0) |
| Urinary tract infection | 62 (10.9) |
| Skin/Soft tissue infection | 43 (7.6) |
| Sepsis and bacteremia | 11 (1.9) |
| Fever of unknown origin/empirical coverage | 166 (29.2) |
| Prophylaxis | 90 (15.8) |
| Multiple infections | 54 (9.5) |
| Not recorded | 16 (2.8) |

**Table S5.A.** Clinical features of patients who received anti-CTLA-4 therapy according to use of antibiotic therapy

| Feature | Antibiotic therapy  (*n* = 270) | No antibiotic therapy  (*n* = 130) | *p* |
| --- | --- | --- | --- |
| IMDC, *n* (%) | 146 (54.1) | 92 (70.8) | 0.002 |
| Mean duration of IMDC symptoms, days (SD) | 40 (198) | 13 (128) | 0.245 |
| Hospitalization, *n* (%) | 105 (71.9) | 44 (47.8) | < 0.001 |
| Mean duration of hospitalization, days (SD) | 8 (7) | 6 (4) | 0.070 |
| ICU admission, *n* (%) | 4 (2.7) | 1 (1.1) | 0.651 |
| Grade of colitis, *n* (%) |  |  | 0.011 |
| 1 | 22 (17.9) | 17 (25.0) |  |
| 2 | 45 (36.6) | 35 (51.5) |  |
| 3 | 53 (43.1) | 13 (19.1) |  |
| 4 | 3 (2.4) | 3 (4.4) |  |
| Grade of diarrhea, *n* (%) |  |  | 0.272 |
| 1 | 35 (24.0) | 25 (27.2) |  |
| 2 | 32 (21.9) | 26 (28.3) |  |
| 3 | 69 (47.3) | 39 (42.4) |  |
| 4 | 10 (6.8) | 2 (2.2) |  |
| Mean duration of steroid administration, days (SD) | 56 (45) | 66 (89) | 0.334 |
| Infliximab/vedolizumab administration, *n* (%) | 41 (28.1) | 21 (22.8) | 0.449 |
| Recurrence of IMDC, *n* (%) | 23 (15.8) | 20 (21.7) | 0.299 |

**Table S5.B.** Clinical features of patients who received anaerobic and aerobic antibiotic therapy among patients who received anti-CTLA-4 therapy. (No. of patients who received antibiotics = 270)

| Feature | Anaerobic  (*n* = 132) | Aerobic  (*n* = 138) | *p* |
| --- | --- | --- | --- |
| IMDC, *n* (%) | 81 (61.4) | 65 (47.1) | 0.021 |
| Immunosuppressive therapy for IMDC, *n* (%) | 65 (49.2) | 44 (31.9) | 0.014 |
| Mean time to IMDC onset, weeks (SD) | 9 (8) | 7 (5) | 0.135 |
| Mean duration of IMDC symptoms, days (SD) | 16 (15) | 71 (296) | 0.096 |
| Hospitalization, *n* (%) | 67 (82.7) | 38 (58.5) | 0.002 |
| Mean duration of hospitalization, days (SD) | 9 (7) | 8 (5) | 0.689 |
| ICU admission, *n* (%) | 4 (4.9) | 0 (0) | 0.129 |
| Grade of colitis, *n* (%) |  |  | 0.009 |
| 1 | 7 (9.6) | 15 (30) |  |
| 2 | 32 (43.8) | 13 (26.0) |  |
| 3 | 31 (42.5) | 22 (44.0) |  |
| 4 | 3 (4.1) | 0 (0) |  |
| Grade of diarrhea, *n* (%) |  |  | 0.571 |
| 1 | 19 (23.5) | 16 (24.6) |  |
| 2 | 15 (18.5) | 17 (26.2) |  |
| 3 | 40 (49.4) | 29 (44.6) |  |
| 4 | 7 (8.6) | 3 (4.6) |  |
| Mean calprotectin level (SD) | 563 (369) | 190 (158) | 0.010 |
| Mean duration of steroid administration, days (SD) | 54 (45) | 59 (46) | 0.641 |
| Infliximab/vedolizumab administration, *n* (%) | 20 (24.7) | 21 (32.3) | 0.356 |
| Recurrence of IMDC, *n* (%) | 12 (14.8) | 11 (16.9) | 0.820 |

**Table S6.A** Clinical features of patients who received anti-PD-1/L1 therapy according to use of antibiotic therapy

| Feature | Antibiotic therapy  (*n* = 299) | No antibiotic therapy  (*n* = 127) | *p* |
| --- | --- | --- | --- |
| IMDC, *n* (%) | 121 (40.5) | 75 (59.1) | 0.001 |
| Mean duration of IMDC symptoms, days (SD) | 21 (45) | 23 (55) | 0.782 |
| Hospitalization, *n* (%) | 63 (52.1) | 19 (25.3) | < 0.001 |
| Mean duration of hospitalization, days (SD) | 7 (8) | 7 (6) | 0.778 |
| ICU admission, *n* (%) | 6 (5.0) | 0 (0.0) | 0.084 |
| Grade of colitis, *n* (%) |  |  | 0.880 |
| 1 | 20 (25.0) | 15 (31.3) |  |
| 2 | 45 (56.3) | 25 (52.1) |  |
| 3 | 12 (15.0) | 6 (12.5) |  |
| 4 | 3 (3.8) | 2 (4.2) |  |
| Grade of diarrhea, *n* (%) |  |  | 0.397 |
| 1 | 42 (34.7) | 27 (36.0) |  |
| 2 | 37 (30.6) | 25 (33.3) |  |
| 3 | 34 (28.1) | 22 (29.3) |  |
| 4 | 8 (6.6) | 1 (1.3) |  |
| Mean duration of steroid administration, days (SD) | 45 (42) | 61 (63) | 0.143 |
| Infliximab/vedolizumab administration, *n* (%) | 11 (9.1) | 10 (13.3) | 0.354 |
| Recurrence of IMDC, *n* (%) | 19 (15.7) | 21 (28.0) | 0.045 |

**Table S6.B** Clinical features of patients who received anaerobic and aerobic antibiotic therapy among patients who received anti-PD-(L)1 therapy. (No. of patients who received antibiotics = 299)

| Feature | Anaerobic  (*n* = 156) | Aerobic  (*n* = 143) | *p* |
| --- | --- | --- | --- |
| IMDC, *n* (%) | 64 (41.0) | 57 (39.9) | 0.906 |
| Immunosuppressive therapy for IMDC, *n* (%) | 37 (23.7) | 27 (18.9) | 0.520 |
| Mean time to IMDC onset, weeks (SD) | 21 (26) | 15 (15) | 0.119 |
| Mean duration of IMDC symptoms, days (SD) | 22 (55) | 21 (32) | 0.848 |
| Hospitalization, *n* (%) | 39 (60.9) | 24 (42.1) | 0.046 |
| Mean duration of hospitalization, days (SD) | 9 (10) | 5 (3) | 0.063 |
| ICU admission, *n* (%) | 6 (9.4) | 0 (0) | 0.029 |
| Grade of colitis, *n* (%) |  |  | 0.172 |
| 1 | 9 (18.8) | 11 (34.4) |  |
| 2 | 27 (56.3) | 18 (56.3) |  |
| 3 | 9 (18.8) | 3 (9.4) |  |
| 4 | 3 (6.3) | 0 (0) |  |
| Grade of diarrhea, *n* (%) |  |  | 0.134 |
| 1 | 19 (29.7) | 23 (40.4) |  |
| 2 | 18 (28.1) | 19 (33.3) |  |
| 3 | 20 (31.3) | 14 (24.6) |  |
| 4 | 7 (10.9) | 1 (1.8) |  |
| Mean duration of steroid administration, days (SD) | 40 (41) | 52 (43) | 0.284 |
| Intravenous steroid administration, *n* (%) | 20 (60.6) | 7 (26.9) | 0.017 |
| Infliximab/vedolizumab administration, *n* (%) | 8 (12.5) | 3 (5.3) | 0.214 |
| Recurrence of IMDC, *n* (%) | 13 (20.3) | 6 (10.5) | 0.210 |
| Colon perforation, *n* (%) | 2 (3.1) | 0 (0) | 0.498 |

**Table S7** Multivariate logistic regression analysis of risk of IMDC

| **Characteristic** | **OR (95% CI)** | ***p*** |
| --- | --- | --- |
| ICI type |  |  |
| Anti-PD-1/L1 | Reference |  |
| Anti-CTLA-4 | 2.16 (1.56-2.99) | < 0.001 |
| Duration of ICI therapy | 1.00 (0.99-1.00) | 0.519 |
| Antibiotic therapy |  |  |
| Anaerobic | 0.44 (0.29-0.66) | < 0.001 |
| Aerobic | 0.34 (0.23-0.51) | < 0.001 |
| None | Reference |  |

*Abbreviations*: *OR* Odds ratio. *CI* Confidence interval

**Table S8** Univariate Cox regression analysis of overall survival in the study population

| **Characteristic** | **HR (95% CI)** | ***p*** |
| --- | --- | --- |
| Age | 1.01 (1.00-1.02) | 0.007 |
| ICI type |  |  |
| Anti-PD-1/L1 | Reference |  |
| Anti-CTLA-4 | 0.72 (0.57-0.91) | 0.005 |
| Combination | 0.65 (0.46-0.91) | 0.012 |
| Stage IV cancer | 1.57 (1.04-2.37) | 0.031 |
| Time to IMDC onset | 0.97 (0.95-0.99) | 0.001 |
| IMDC | 0.44 (0.36-0.55) | <0.001 |
| Duration of IMDC symptoms | 0.99 (0.99-0.99) | 0.007 |
| Calprotectin level | 1.00 (1.00-1.01) | 0.006 |
| Antibiotic therapy | 2.21 (1.70-2.86) | <0.001 |
| Anaerobic | 1.54 (1.22-1.96) | <0.001 |
| Timing of antibiotic therapy |  |  |
| Before ICI therapy | Reference |  |
| After ICI therapy | 1.64 (1.11-2.38) | 0.013 |

*Abbreviations*: *HR* Hazard ratio. *CI* Confidence interval

**Fig. S1.** Kaplan-Meier curves for overall survival of patients who did and did not receive antibiotic therapy.

**Fig. S2.** Kaplan-Meier curves for overall survival of patients who did and did not receive antibiotic therapy with antianaerobic activity.
